# Orthostatic Tremor Is Evoked by Muscle Load Without the Need for Orthostatic Position

**DOI:** 10.1002/mdc3.70270

**Published:** 2025-08-01

**Authors:** Karolina af Edholm, Gonzalo Uribarri, Mathias Sundgren, Anders Svenningsson, Erik Fransén

**Affiliations:** ^1^ Department of Clinical Sciences Karolinska Institutet Danderyds Sjukhus Stockholm Sweden; ^2^ School of Electrical Engineering and Computer Science KTH Royal Institute of Technology Stockholm Sweden; ^3^ Digital Futures KTH Royal Institute of Technology Stockholm Sweden; ^4^ Science for Life Laboratory KTH Royal Institute of Technology Stockholm Sweden; ^5^ Department of Clinical Neuroscience Karolinska Institutet Stockholm Sweden; ^6^ Department of Neurology Karolinska University Hospital Stockholm Sweden

**Keywords:** muscle load, orthostatic position, primary orthostatic tremor

## Abstract

**Background:**

Primary orthostatic tremor (POT) is a rare movement disorder characterized by a high‐frequency tremor and a considerable feeling of unsteadiness. People with POT are significantly affected in their daily activities and have reduced quality of life. The tremor occurs in standing position and dominates in the lower extremities. However, whether an orthostatic position is essential for evoking the tremor has not been fully clarified.

**Objectives:**

To investigate how POT is responding to muscle load in non‐orthostatic positions.

**Methods:**

In this work, we measured the tremor in POT patients using a smartphone running a tremor analysis application attached to the proximal fibula in supine, seated and standing position, as well as when they were subjected to muscle load in a leg press machine in a seated position.

**Results:**

We demonstrate that the tremor can be elicited by muscle load while the patient is sitting, compatible with a weight‐bearing isometric tremor, without the need to be in standing position. Furthermore, the weight of the load modulates the amplitude of the elicited tremor, but not its frequency.

**Conclusion:**

These findings suggest that POT is a weight‐bearing hyperkinetic disorder and challenge the conventional assumption that the POT patient must be in standing position for the tremor to fully manifest. The results potentially have implications for understanding the mechanisms underlying POT, and can be of importance in future experimental studies, for example MRI, when standing position is not an available option.

Primary orthostatic tremor (POT) is a rare, progressive neurological disease, defined as an isolated tremor syndrome characterized by a generalized tremor that occurs while standing, at a frequency ranging from 13 to 18 Hz.^1^ POT is slightly more prevalent in women and symptoms usually begin after the age of 50 and gradually worsen over time. Diagnosis is often delayed for several years due to difficulties in recognizing the condition.^2^ Patients with POT experience an intense sensation of imbalance commonly accompanied by fatigue and pain. This often severely limits their ability to remain in standing position and has a negative effect on their daily activities and quality of life.^3^ The high frequency of the tremor (13–18 Hz) is characteristic for POT and the individual frequency remains stable over time in the patient.^4^ The tremor is typically coherent bilaterally in the leg and axial muscles when activated in standing position, and patients report disappearance or at least a greatly diminished perception of the tremor when sitting or walking.^5^, ^6^ Although the exact pathophysiology of POT is still unknown, the prevailing hypothesis argues that POT is caused by a disturbance from a central oscillator or a disruption in the cerebello‐brainstem‐thalamo‐cortical network of postural control, explaining the high coherence of tremor activity bilaterally and the alternating activation of muscle agonists and antagonists.^6^

There are contradicting results in the earlier studies investigating the triggering factor for evoking tremor in patients with POT, with some suggesting the orthostatic position itself as the primary cause and other studies emphasizing the role of muscle load.^7^, ^8^, ^9^ There are some previous reports of the appearance of a fast synchronous tremor of 13–18 Hz in positions other than standing in a minority of patients with POT.^2^, ^5^, ^7^, ^8^, ^10^, ^11^, ^12^, ^13^ However, whereas these small numbers of observations have been reported, the focus has remained on the orthostatic position as the defining characteristic of POT. Thus, whether POT depends on the orthostatic position or is primarily a weight‐bearing hyperkinetic disorder of isometric muscle tension remains an open question.

In the present study, we investigated the tremor in seven patients with POT to determine whether a tremor of the same fast frequency observed during standing (13–18 Hz) could be induced by isometric muscle contraction independently of orthostatic position, and whether the level of muscle force plays a role in tremor generation. Tremor was recorded with a smartphone application attached to the leg. Smartphone applications that utilize the accelerometer for measuring movements have recently emerged as a convenient bedside tool for identifying tremor frequency.^14^, ^15^, ^16^, ^17^

## Methods

### Cohort Description

Patients with POT were recruited from the cohort of the Study of Orthostatic Tremor in Sweden (SOTIS). Seven participants without clinically significant orthopedic conditions or neuropathy in the lower extremities were invited to participate. All participants were diagnosed with POT according to the Consensus Statement of the Classification of Tremors (Bhatia et al).^1^ Participants were allowed to take their regular medications at the day of the examination. The study was approved by the Swedish ethical review authority (Etikprövningsmyndigheten, ref. no 2019‐01490 and 2022‐01802‐02), and all participants provided written informed consent. The study was conducted in accordance with the Good Clinical Practice guidelines and the principles of the Declaration of Helsinki.

### Experimental Protocol

**Figure 1:**
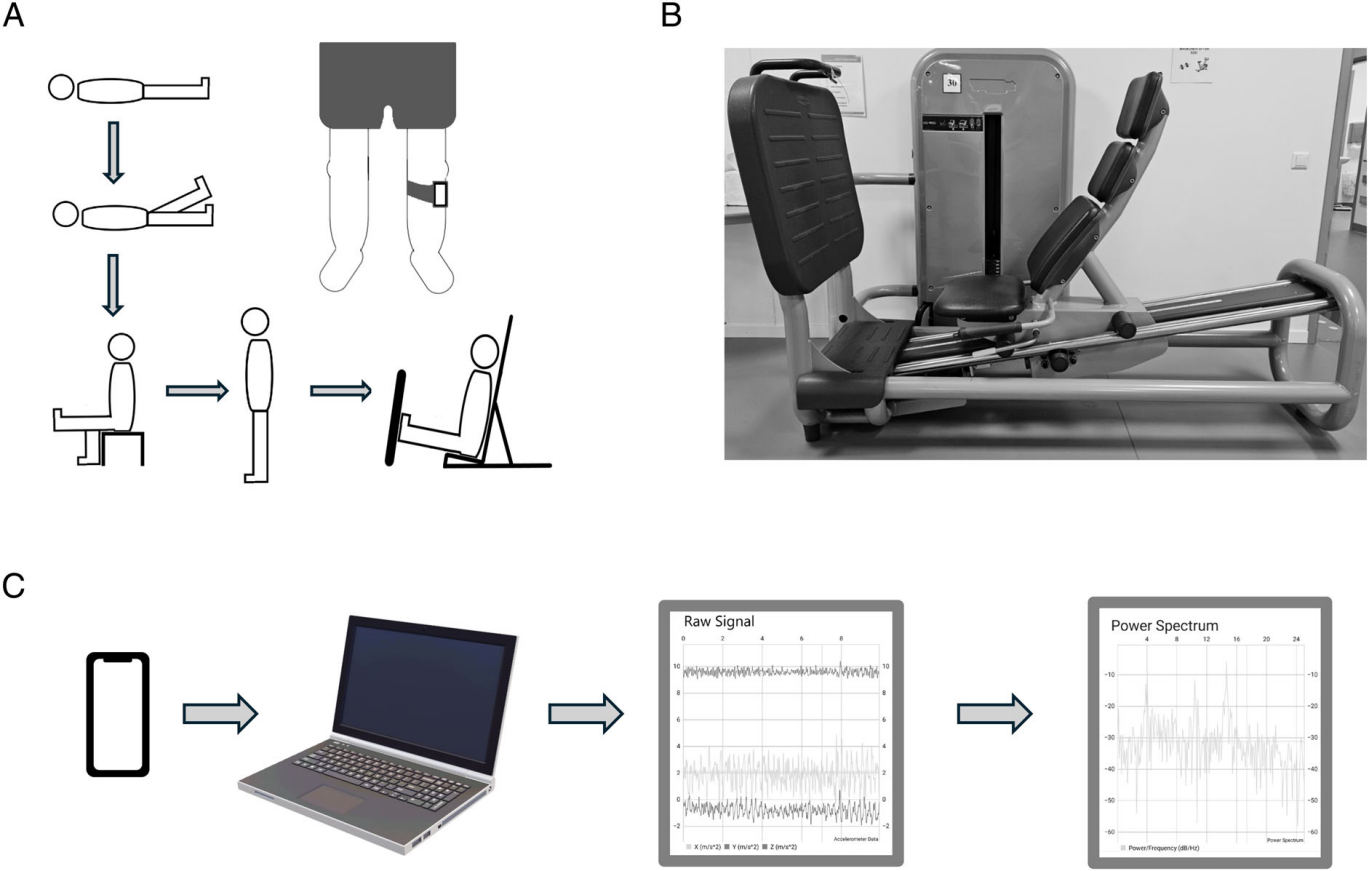
Experimental setup. (A) Order of positions investigated. A smartphone running the Vibra App was attached to the patient's proximal fibula using a sports armband. (B) Leg press machine used in the experiment. (C) A systematic presentation of the recording process.

Tremor movement was measured with a smartphone application. The smartphone was attached to the participants’ proximal fibula with a sports armband. All participants were initially examined while lying down and relaxing, then lying down with one leg lifted approximately 20 to 40 degrees, sitting up with an extended knee, and then while standing up (Fig. 1). Tremor activity was examined in both the right and left leg, with each measurement lasting 10 s.

In the second phase, participants were sitting in a leg press machine (Technogym Leg Press MB500N0‐ALVOGGGP), with seating adjusted for individual comfort. A leg press machine allows the application of a defined load to the leg while the balance and postural control required for standing is not engaged. Participants maintained a fixed isometric contraction while measurements were recorded, incremental loads were applied between test sessions, starting at 10 kg and increasing gradually to the patient's tolerance limit or up to 100 kg.

All participants were instructed to fully stretch both their legs straight and maintain this position while tremor measurements took place. Every measurement was 10 s long and was done on the left leg. After each measurement participants were allowed to rest, and at subsequent measurement, extra weight had been added to the machine. All participants started with the lowest weight of 10 kg, and then 10 kg was added at every subsequent step. Participants were allowed to terminate the procedure if they found the weight too heavy. No participant was examined with weights exceeding 100 kg.

### Data Acquisition

Tremor data was measured with the accelerometer in a smartphone Xperia 10 Sony Mobile running the application Vibra. The smartphone was attached to the participants’ proximal fibula with a sports armband (Streetz sport, Reflective sports armband). Data was registered with the smartphone application Vibra (https://github.com/krisgun/Vibra). The smartphone application Vibra was developed by Kristoffer Gunnarsson at the KTH Royal Institute of Technology (Kungliga Tekniska Högskolan) in Stockholm, Sweden (https://www.diva-portal.org/smash/get/diva2:1750950/FULLTEXT01.pdf) where the validation of the accuracy of measurement with Vibra was confirmed in comparison to EMG. The technical functionality allowed a integer sampling frequency of 0–999 Hz. During measurements, a sampling frequency of 200 Hz was chosen.

### Data Analysis

After acquisition, the accelerometer data was processed using the Python programming language. As a first step, the magnitude of the acceleration vector, *m*(*t*), was calculated to provide a scalar representation of the tremor dynamics. The magnitude was computed as:

$$m\left(t\right)=\surd \left(x{\left(t\right)}^{2}+y{\left(t\right)}^{2}+z{\left(t\right)}^{2}\right)$$

where *x*(*t*), *y*(*t*), and *z*(*t*) correspond to the acceleration components measured along the three orthogonal axes of the device, expressed in meters per second squared (m/s^2^). By deriving the magnitude of the acceleration, we eliminated directional dependencies, ensuring that the analysis captured the overall intensity of the tremor and was not biased by movement along any axis.

Then, a fourth‐order Butterworth bandpass filter was applied to the magnitude signal m(t) using the SciPy library, with cutoff frequencies set at 10 Hz (low cut) and 22 Hz (high cut). This filtering step effectively preserved the frequency range characteristic of orthostatic tremor (13–18 Hz) (Bhatia et al, 2018), while attenuating unwanted low and high frequency noise and artifacts. Frequencies below 10 Hz, which primarily contain motion artifacts and slow postural adjustments, were thus suppressed, while frequencies above 22 Hz, which are often dominated by high‐frequency sensor noise and external mechanical vibrations, were also removed.

The noise profile of the accelerometer signal had a typical 1/f distribution, meaning that the noise amplitude was higher at lower frequencies. This further justified the use of a bandpass filter to ensure that low frequency signal artifacts did not obscure the tremor‐related components in the 13–18 Hz range.

The power spectrum of the filtered signals was subsequently computed using NumPy's Fast Fourier Transform (FFT) implementation. From the resulting power spectrum, the characteristic tremor frequency was determined as the frequency corresponding to the peak power value, which represented the most prominent oscillatory component within the 10–22 Hz range.

To quantify the dominance of the tremor in the measured signal, we computed the tremor magnitude. This was done by summing the spectral power within a 0.6 Hz window centered on the identified peak frequency and dividing it by the total signal power, providing a normalized measure of tremor dominance. A magnitude value close to one indicates that the identified frequency peak accounts for most of the signal's energy, suggesting a clearly detectable tremor. In contrast, low magnitude values suggest the absence of a tremor, indicating that the detected peak is likely part of background noise. This approach ensured a robust characterization of the spectral prominence of the tremor, while allowing for small frequency variations due to physiological and sensor‐related factors.

## Results

### Clinical Parameters

Seven patients were recruited to the study. All participants had EMG verified orthostatic tremor in standing position between 14 and 16 Hz and no tremor in resting (supine) position. Average age was 70.3 (± 10.2) years and disease duration 8.8 (± 4.8) years. Average body weight was 75.1 (± 13.3) kg and average Body Mass Index was 25.0 (± 2.8). Four males and three females were included. Three participants were treated with gabapentin, one with propranolol, one with clonazepam, and two participants had no treatment. Clinical data is presented in Table 1.

**TABLE 1:** Clinical and demographic characteristics of patients with POT

Patient	Age (years)	Sex	Weight (kg)	Disease duration (years)	Tremor Frequency (Hz)	Medication
1	79	M	77	7	14.31	None
2	82	M	98	11	14.89	Gabapentin 1200 mg 3 times a day
3	72	F	55	4	15.31	Gabapentin 200 mg 2 times a day
4	51	F	66	2	16.00	None
5	74	M	78	7	15.41	Clonazepam 0.5 mg 2 times a day
6	66	F	72	11	14.40	Gabapentin 200 mg once a day
7	68	M	80	16	14.24	Propranolol 10 mg 3 times a day
Average	70.3		75.1	8.3	14.94	
StD	10.2		13.3	4.8	0.67	

### Tremor in Supine, Seated and Standing Position

All seven patients were examined by principal investigator K. af Edholm in supine, seated and standing position. No patient presented tremor while relaxing in supine position nor when lifting one leg 20–40 degrees during supine position (Figs. S1 and S2). When seated with a fully extended knee one patient (patient 7) presented a visible tremor peak in the left leg in the same frequency range as when standing (Fig. S3), no tremor was visible when the right leg was lifted (data not shown).

**Figure 2:**
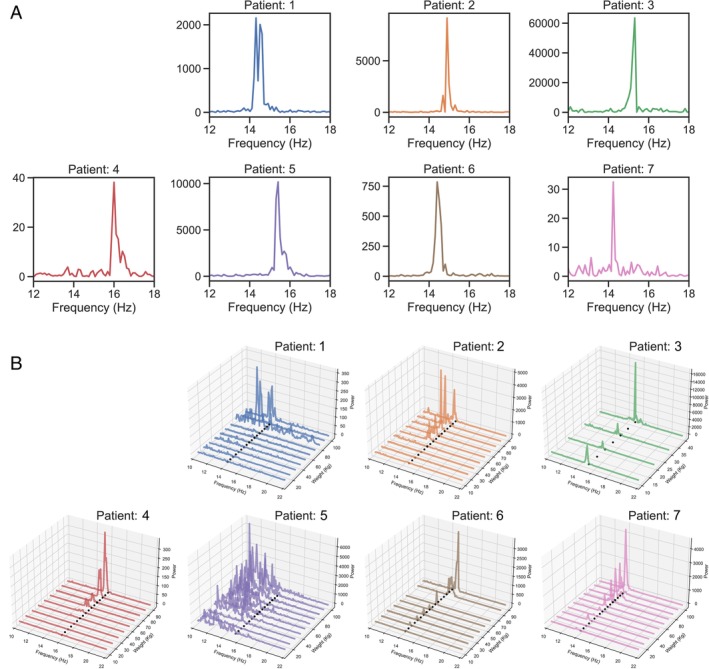
(A) Power spectra of tremor activity during standing. (B) Power spectra of tremor activity in response to gradual muscle load. The three axes represent frequency (Hz), power amplitude of the tremor, and gradual increase of weight load (kg). Dotted line: individual tremor frequency observed when standing.

**Figure 3:**
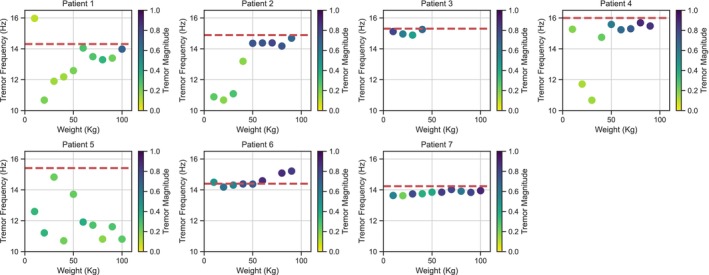
Tremor frequency and magnitude as a function of load. Magnitude of tremor is encoded by color (darker shades indicate a more dominant tremor). X‐axis: weight load (kg). Y‐axis: tremor frequency (Hz). Red dashed line: tremor frequency in standing (see Fig. 2).

All seven patients presented a fast tremor activity (14–16 Hz) immediately when standing up (Table 1). The raw power spectra corresponding to the measurements recorded during the standing position for the seven patients are presented in Fig. 2A. For all seven patients, a well‐defined and prominent peak is observed within the frequency range of 14 to 16 Hz, consistent with the characteristic high‐frequency tremor associated with POT. The frequency of this peak, referred to as the standing tremor frequency, serves as a baseline for comparisons in other experimental conditions (Figs. 2B and 3).

### Tremor in Leg Press Machine

Power spectrum analysis of data from the leg press machine showed that 6 of 7 patients presented minimal tremor activity at lighter loads and then displayed a sharp increase in tremor power at higher weights, suggesting load‐sensitive modulation of tremor intensity (Fig. 2B). Specifically, for five of the seven patients (patient 2, 3, 4, 6, 7), a distinct load threshold can be observed, beyond which tremor activity is consistently elicited. In these cases, the emergence of a tremor peak occurs as the applied load exceeds this threshold, highlighting the load‐dependency of tremor onset. Three of the patients (1–3) displayed a variable amplitude of tremor after onset, however, any correlation between tremor amplitude and increase of muscle force above the necessary level for resistance of the weight load could not be explored by this method. In patient 4, 6 and 7 the power amplitude of the tremor increases with higher loads, reflecting a positive modulation of tremor intensity by muscle force. Furthermore, patient 5 exhibited no observable tremor response across the load range, suggesting either a higher activation threshold (exceeding the 100 kg maximum weight used), an effect of clonazepam, or a distinct tremor dynamic unique to this individual.

We further analyzed the data from the leg press experiments. In Fig. 3, we can see that well‐defined tremor peaks are concentrated around the standing tremor frequency. This consistency suggests that once the tremor is triggered by sufficient muscle load, notably it oscillates at a frequency close to that observed when the patient is standing. The gradual appearance of darker points as the load increases highlights the load‐dependent nature of tremor initiation and its modulation by muscle force. This modulation effect is also apparent in Fig. S4, which shows the height of the frequency peak as a function of load. As the weight increases, the peak height grows, indicating that the amplitude of the tremor increases with greater muscle load.

Patient's body weight presented a positive but not significant correlation to the weight of the tremor threshold (spearman correlation = 0.39, *p*‐value = 0.43). Neither did we notice any latency of tremor onset in any of the measurements.

## Discussion

Our results demonstrate that the occurrence of the characteristic high‐frequency tremor in POT patients may be elicited by isometric muscle force under external pressure from a leg press machine without the patient being in an orthostatic standing position. Moreover, we found that the power amplitude of the tremor increased with higher loads, reflecting a positive modulation of tremor intensity by muscle force. Furthermore, the tremor is often not induced until a significant weight is introduced. Lifting the leg against gravity in a seated or supine position does not seem to engage enough muscle force to induce the tremor in the majority of patients with POT (6 out of our 7 patients). Our observations support the hypothesis that weight‐bearing isometric muscle force is a key factor in tremor generation and modulation in POT, challenging the conventional view of the orthostatic position as a necessary trigger. These findings could be of value for future studies, enabling examinations of POT in positions other than standing, including experimental settings such as imaging modalities where upright posture is not feasible.

To the best of our knowledge, this is the first study to demonstrate that POT tremor can be elicited in the sitting position with weight‐load applied in most patients and to clearly show that its onset is modulated by muscle load. Importantly, these novel findings align with previous reports indicating that some POT patients can exhibit the same high‐frequency tremor in positions other than standing. There have been previous reports of high frequency tremor in other positions than standing, but these have been either just descriptions of a single or a few cases or shown in a minority of patients in larger groups, leaving the question of why the tremor might be elicited by weight‐bearing conditions unanswered. When the symptoms of POT were first described by Pazzaglia et al in 1970 they also investigated whether the tremor was depending on orthostatic position by tilting one patient from lying to an erected position, and found that this intervention was not enough to elicit tremor.^18^ Later in 1989, Uncini and colleagues found in one POT patient that the tremor was induced in a sitting position when the patient pressed the soles of the feet to the floor intending to stand up.^10^ Moreover, Walker and colleagues presented a case report of one POT patient with tremor in a supine position with feet pressing against a wall.^11^ Furthermore, Köster and colleagues found a fast synchronized stable tremor activity in all isometrically contracted muscles of the face, neck, trunk and limbs independently of a standing, seated or supine position in six patients.^12^ In 1999, Boroojerdi and colleagues investigated six patients with POT using a harness system to manipulate weight‐bearing conditions.^8^ They found that all six patients exhibited fast, synchronized tremor while in weight‐bearing positions, such as standing upright or on all fours. However, when the harness supported the patients, eliminating the weight‐bearing load, the orthostatic position alone did not induce tremor. Furthermore, five of these patients also demonstrated tremor in isometrically contracted limb muscles while lying in a supine position.^8^ McManis and colleagues found that six out of 28 POT patients developed the same fast tremor activity during forceful isotonic or isometric muscle contractions in non‐weight‐bearing movements, and one additional patient even developed the same fast tremor activity in postural activation of upper limb muscles.^13^ Hassan and colleagues also found a postural tremor at the same fast tremor frequency in a minority of POT patients (three out of 16 examined patients).^9^ All these results indicate that at least in a part of the patients with POT the tremor is mainly dependent on isometric muscle activation and that the orthostatic position is of less importance. Diversity in findings could reflect a variability in phenomenology among POT patients. It could, however, also be a consequence of methodological differences, for example the level of the muscle load used, or the severity of the disease among the examined participants. In particular, our results of individual thresholds for the induction of tremor might explain why some patients in the studies discussed above did not show tremor.

The importance of muscle load was contradicted by the results of Spiegel and colleagues (2006).^7^ They examined seven POT patients with an experimental setup using both weight‐bearing relief and tilting, to explore the impact of muscle contraction and orthostatic position. They found that weight‐bearing relief did not significantly affect tremor intensity, and that tremor still prevailed unchanged when patients were lifted with a crane, while tilting from orthostatic position to supine did significantly lower tremor intensity or made it disappear completely.^7^ These results speak in favor of the theory that the orthostatic position is of greater importance than muscle force in evoking the tremor activity, but does not exclude the possibility that muscle activation still has an important role in generation of tremor since they did not quantify the level of muscle contraction in the different examined body positions.

The Consensus Statement on the Classification of Tremors,^1^ defines POT as an isolated tremor syndrome that occurs in standing. Instead, we agree with Hassan and van Gerpen^9^ that POT should be viewed as a weight‐bearing hyperkinetic disorder where the nomenclature “orthostatic” may be useful in the literature but does not fully cover its phenomenology. Even if the tremor generates a minimal movement of the limb, the intention of the posture is isometric, and we hypothesize that POT is a disorder of weight‐bearing isometric muscle activation. This clarification could be of importance for patients experiencing tremor in other positions than standing.

### Limitations

The main limitation of our study is the small number of participants, which is an inherent scientific obstacle of rare disorders.

The use of a smartphone application offers a convenient and accessible method for capturing the movement of the body part it is attached to but does not provide information about the activity of the muscles. Electromyography (EMG) remains the gold standard for confirming tremor frequency, assessing bilateral coherence, and diagnosing the condition. In our study, we did not directly monitor muscle force using EMG across the different test positions, which may explain why Patient 7 exhibited tremor activity only in the left leg while seated with an extended knee. Incorporating EMG would have provided more detailed insights into the muscle‐specific response to load. In this study we did not assess tremor severity and cannot exclude a correlation between tremor weight threshold and symptom severity. A future study with a larger cohort including rating scales like the Orthostatic Tremor Severity and Disability (OT‐10) scale or Orthostatic Tremor Impact Profile (OTIP) scale could further expand the knowledge of POT. We did not explore if there is a tremor threshold of the upper extremities, this could also be of interest for future studies.

## Conclusion

The results of our study support the hypothesis that tremor in POT patients may be evoked by load‐dependent isometric muscle activity without the need of the orthostatic position. The muscle load required to elicit tremor differed between individuals, and once this threshold was exceeded, increasing load weight modulated tremor amplitude but did not affect tremor frequency. Future studies including larger numbers of patients and exploring tremor responses to different weight‐bearing conditions and body positions are needed to confirm these results.

## Author Roles

(1) Research project: A. Conception, B. Organization, C. Execution; (2) Statistical Analysis: A. Design, B. Execution, C. Review and Critique; (3) Manuscript Preparation: A. Writing of the first draft, B. Review and Critique.

K.E.: 1A, 1B, 1C, 2A, 2C, 3A, 3B.

G.U.: 1A, 2A, 2B, 2C, 3A, 3B.

M.S.: 1A, 2C, 3B.

A.S.: 1A, 2C, 3B.

E.F.: 1A, 2A, 2C, 3B.

## Disclosures

**Ethical Compliance Statement:** The study was approved by the Swedish Ethical Review Authority (Etikprövningsmyndigheten, ref. no 2019‐01490 and 2022‐01802‐02). All participants provided written informed consent in accordance with the Declaration of Helsinki. We confirm that we have read the Journal's position on issues involved in ethical publication and affirm that this work is consistent with those guidelines.

**Funding Source and Conflict of Interest:** This study has been financed by a private donation, and by fundings from the Promobilia Foundation and the dBrain grant from KTH Digital Futures. The authors declare that there are no conflicts of interest relevant to this work.

**Financial Disclosure for All Authors (for the Preceding 12 Months):** KaE: Employment as consultant in neurology at the neurological clinics of Danderyds Hospital and Neuroenheten Sabbatsberg, Stockholm, Sweden. GU: Employment as postdoctoral researcher at the KTH Royal Institute of Technology, Stockholm, Sweden. Support from KTH DigitalFutures, and grant from Promobilia Foundation A23122. MS: Employment as senior consultant in neurology at Karolinska University Hospital and Academic Specialist Center in Stockholm, Stockholm, Sweden. AS: Employment as professor of Neurology at Karolinska Institutet Danderyd hospital and consultant in neurology at the neurological clinic of Danderyds hospital, Stockholm, Sweden. Support from Swedish Research Council grants 2016‐00398 and 2020‐00229. EF: Employment as professor at the KTH Royal Institute of Technology, Stockholm, Sweden. Support from Swedish VR grant 2022‐01079, Swedish Vinnova grant 2024‐00247, Swedish NAISS 2024/5‐149 and 2024/6‐6‐78, KTH DigitalFutures.

## Supporting information


**Figure S1.** Power spectra of measurements from the left leg while participants were lying down in a relaxed position. Dotted line: tremor frequency (Hz) in standing.

**Figure S2.** Power spectra of measurements from the left leg while participants were lying down with the left leg lifted 20–40 degrees. Dotted line: tremor frequency (Hz) in standing.

**Figure S3.** Power spectra of measurements from the left leg while participants were sitting up with one leg extended. Dotted line: tremor frequency (Hz) in standing.

**Figure S4.** Tremor peak height and magnitude as a function of load. Magnitude of tremor is encoded by color (darker shades indicate a more dominant tremor). X‐axis: weight load (kg). Y‐axis: tremor peak height (a.u.).

## Data Availability

Data is available for sharing upon request by contacting corresponding authors.
