# Supplementary figures and images for: Orthostatic Tremor Is Evoked by Muscle Load Without the Need for Orthostatic Position
Source: Mov Disord Clin Pract. 2025 Aug 1;13(1):208–15. doi: 10.1002/mdc3.70270 (PMC12839475; doi:10.1002/mdc3.70270)

# Left Leg Rest

Patient: 1

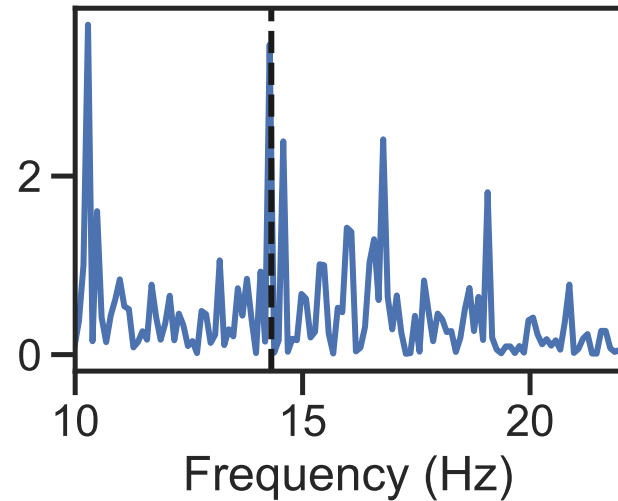

Patient: 2

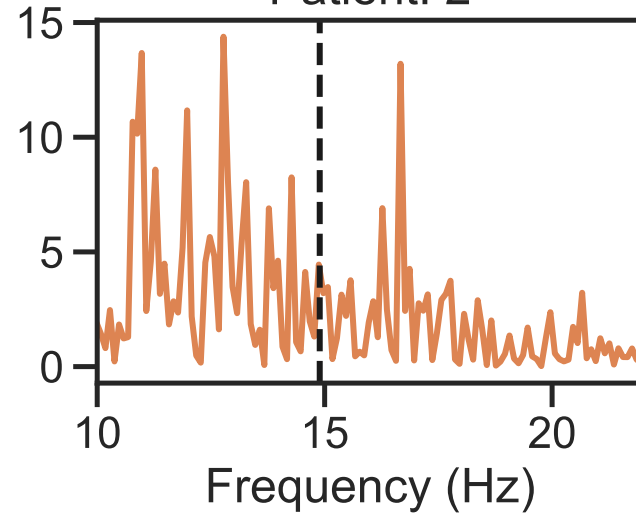

Patient: 3

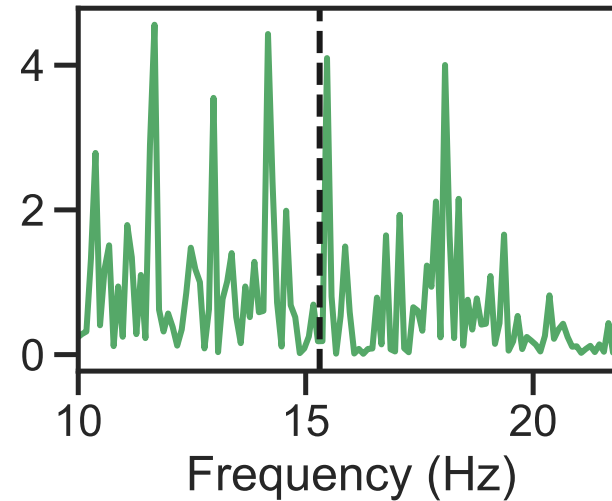

Patient: 4

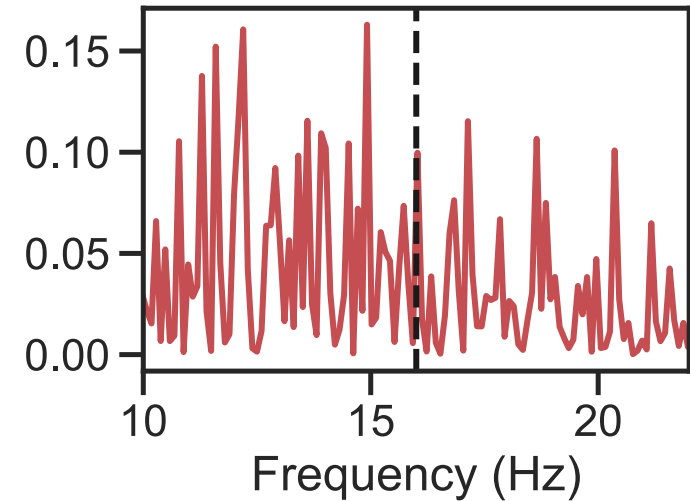

Patient: 5

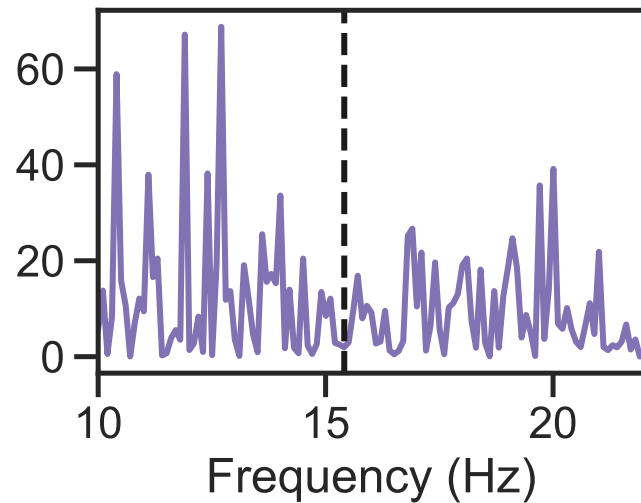

Patient: 6

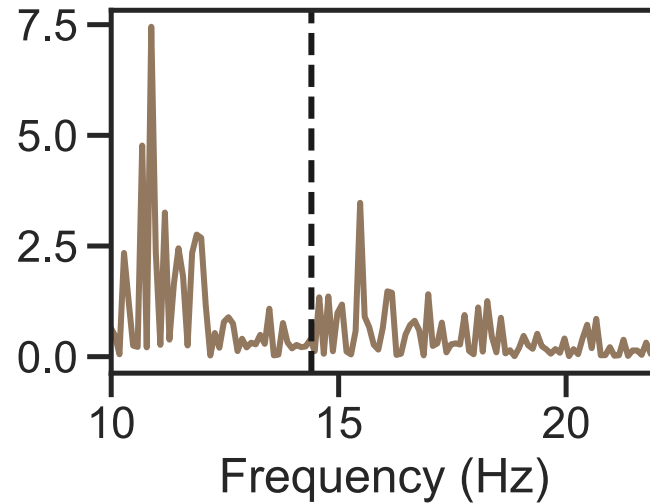

Patient: 7

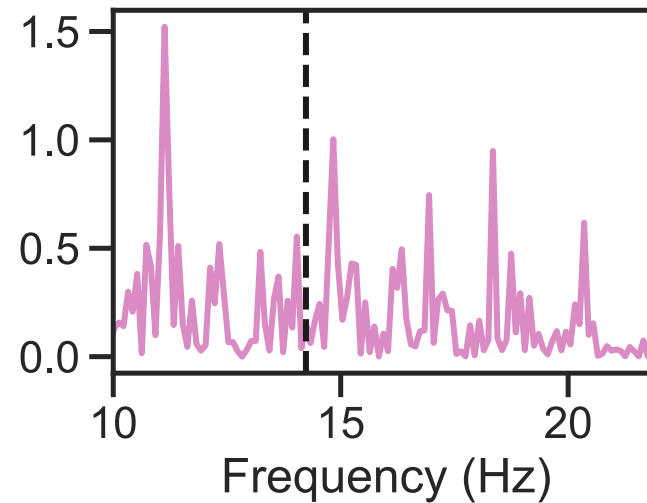

Supplement: Supplementary file 1 — Figure S1. Power spectra of measurements from the left leg while participants were lying down in a relaxed position. Dotted line: tremor frequency (Hz) in standing. [file MDC3-13-208-s001.pdf]

# Left Leg Lifted

Patient: 1

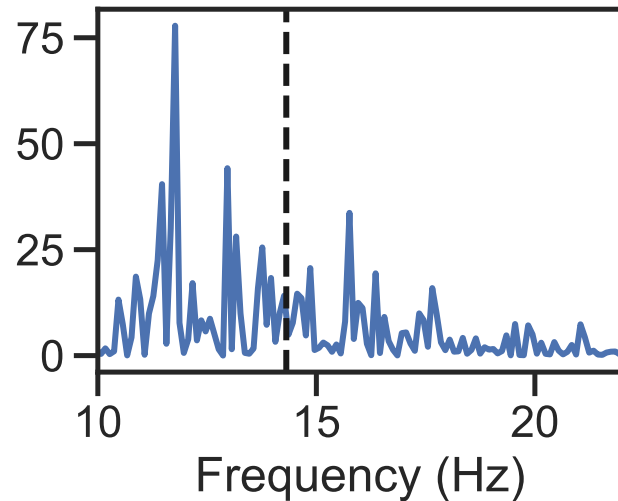

Patient: 2

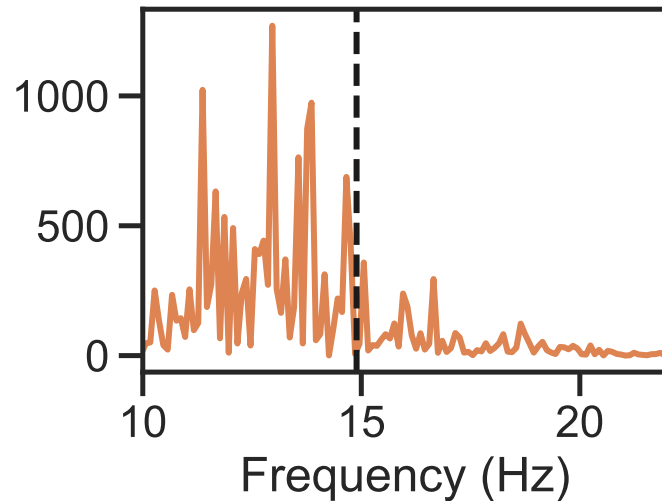

Patient: 3

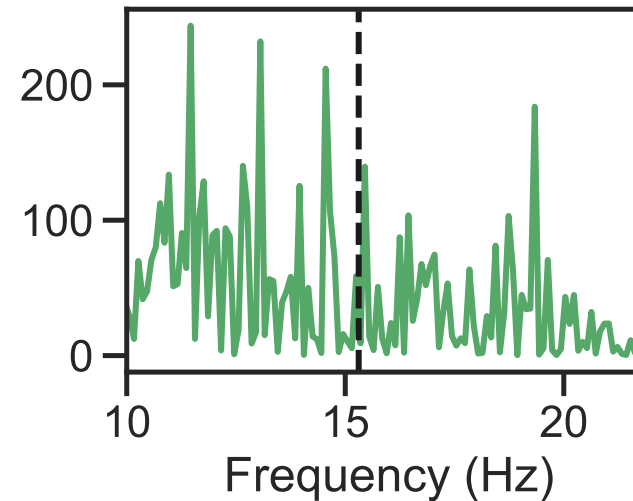

Patient: 4

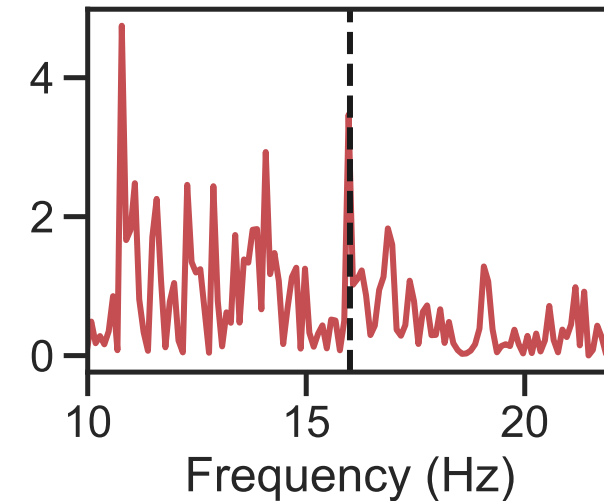

Patient: 5

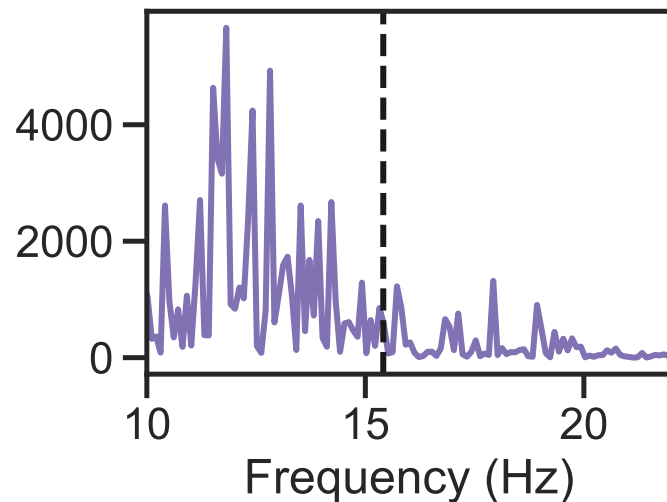

Patient: 6

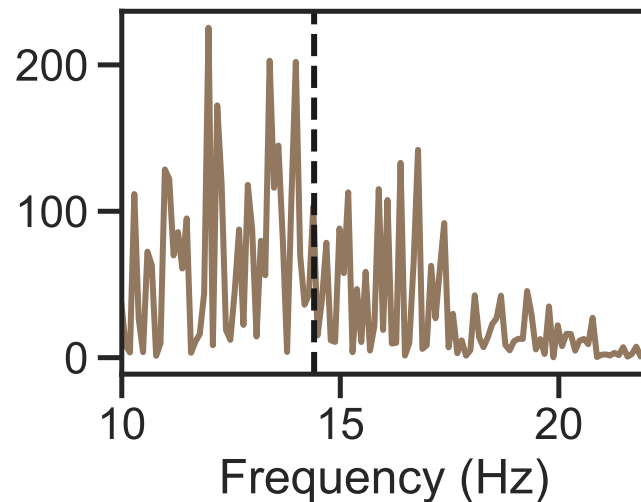

Patient: 7

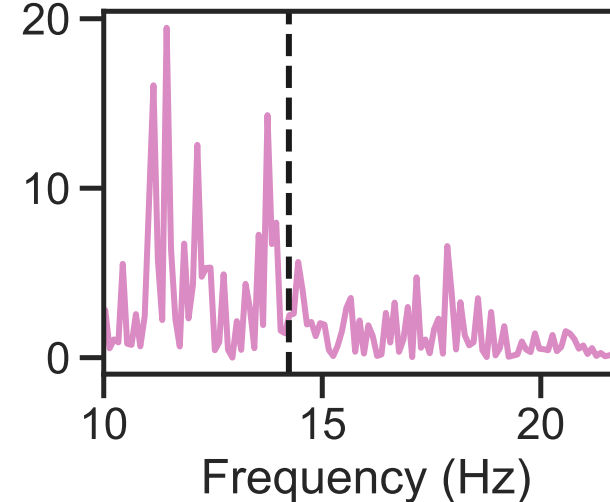

Supplement: Supplementary file 2 — Figure S2. Power spectra of measurements from the left leg while participants were lying down with the left leg lifted 20–40 degrees. Dotted line: tremor frequency (Hz) in standing. [file MDC3-13-208-s004.pdf]

# Left Leg Extension

Patient: 1

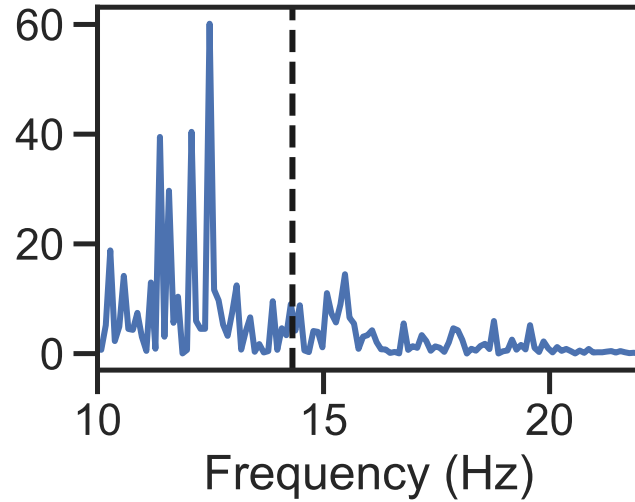

Patient: 2

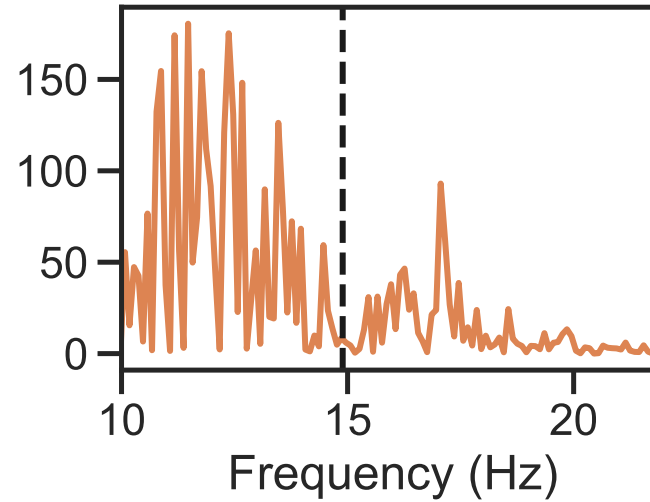

Patient: 3

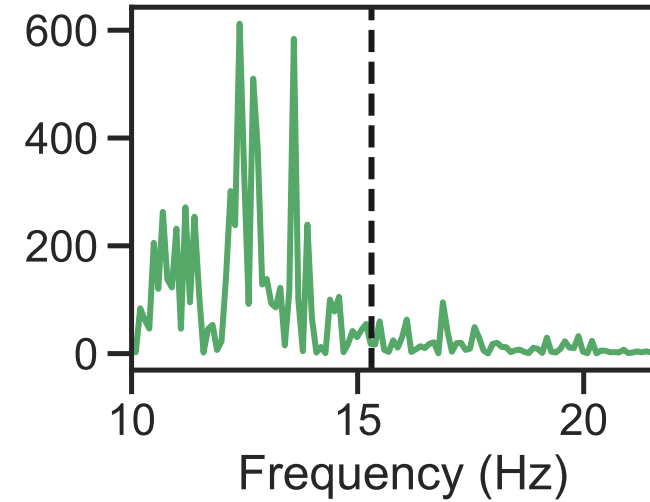

Patient: 4

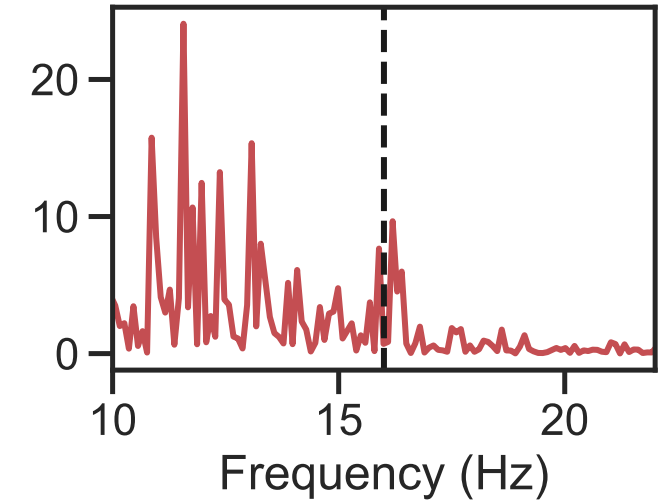

Patient: 5

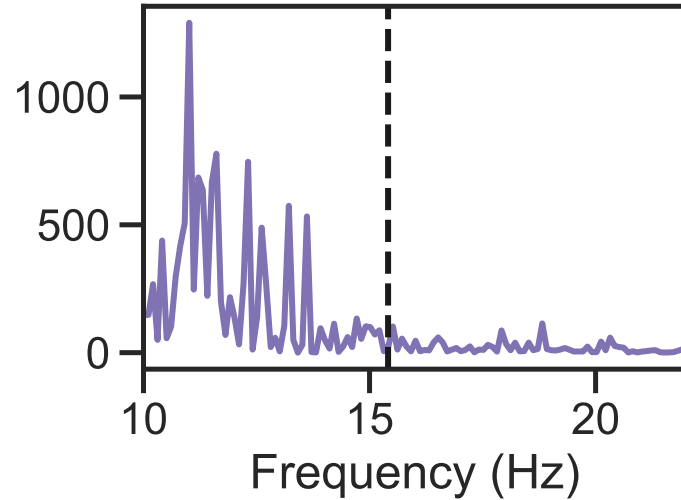

Patient: 6

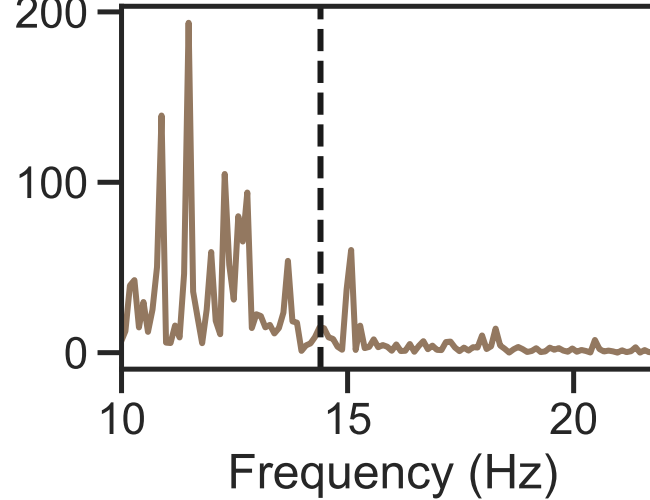

Patient: 7

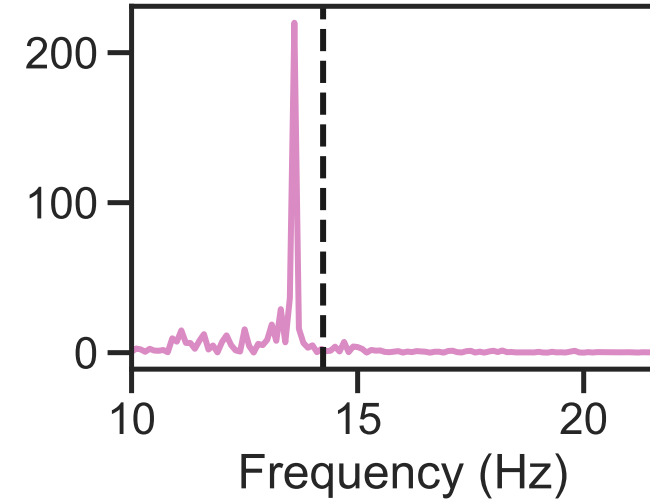

Supplement: Supplementary file 3 — Figure S3. Power spectra of measurements from the left leg while participants were sitting up with one leg extended. Dotted line: tremor frequency (Hz) in standing. [file MDC3-13-208-s002.pdf]

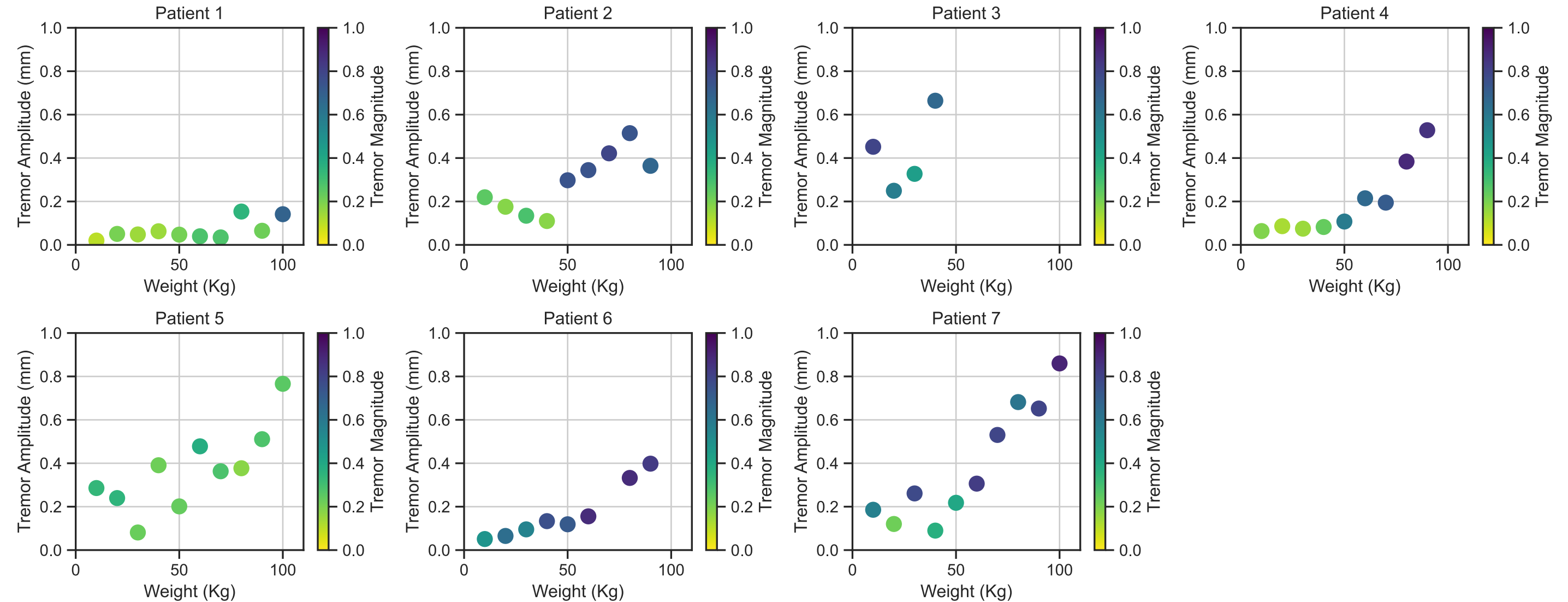

Supplement: Supplementary file 4 — Figure S4. Tremor peak height and magnitude as a function of load. Magnitude of tremor is encoded by color (darker shades indicate a more dominant tremor). X‐axis: weight load (kg). Y‐axis: tremor peak height (a.u.). [file MDC3-13-208-s003.pdf]
